# CtpB is a plasma membrane copper (I) transporting P-type ATPase of *Mycobacterium tuberculosis*

**DOI:** 10.1186/s40659-020-00274-7

**Published:** 2020-02-13

**Authors:** Andrés León-Torres, Epifania Arango, Eliana Castillo, Carlos Y. Soto

**Affiliations:** Chemistry Department, Faculty of Sciences, Universidad Nacional de Colombia, Carrera 30 N° 45-03, Ciudad Universitaria, Bogotá, Colombia

**Keywords:** *Mycobacterium tuberculosis*, P-type ATPase, CtpB, Plasma membrane, Copper transport

## Abstract

**Background:**

The intracellular concentration of heavy-metal cations, such as copper, nickel, and zinc is pivotal for the mycobacterial response to the hostile environment inside macrophages. To date, copper transport mediated by P-type ATPases across the mycobacterial plasma membrane has not been sufficiently explored.

**Results:**

In this work, the ATPase activity of the putative *Mycobacterium tuberculosis* P_1B_-type ATPase CtpB was associated with copper (I) transport from mycobacterial cells. Although CtpB heterologously expressed in *M. smegmatis* induced tolerance to toxic concentrations of Cu^2+^ and a metal preference for Cu^+^, the disruption of *ctpB* in *M. tuberculosis* cells did not promote impaired cell growth or heavy-metal accumulation in whole mutant cells in cultures under high doses of copper. In addition, the Cu^+^ ATPase activity of CtpB embedded in the plasma membrane showed features of high affinity/slow turnover ATPases, with enzymatic parameters *K*_*M*_ 0.19 ± 0.04 µM and *V*_*max*_ 2.29 ± 0.10 nmol/mg min. In contrast, the *ctpB* gene transcription was activated in cells under culture conditions that mimicked the hostile intraphagosomal environment, such as hypoxia, nitrosative and oxidative stress, but not under high doses of copper.

**Conclusions:**

The overall results suggest that *M. tuberculosis* CtpB is associated with Cu^+^ transport from mycobacterial cells possibly playing a role different from copper detoxification.

## Background

Tuberculosis (TB) is an infectious disease caused by the acid-fast bacillus *Mycobacterium tuberculosis*, which in turn is the origin of one of the biggest public health problems worldwide [1]. According to the World Health Organization (WHO), there were 10.4 million new cases and 1.3 million deaths by TB in 2017 [1]. Currently, vaccination with the Bacillus Calmette-Guerin (BCG) and chemotherapy are the common strategies for TB control. The tuberculous infection begins when mycobacteria is phagocytosed by macrophages and dendritic cells in the pulmonary alveoli [2]. *M. tuberculosis* persists inside macrophages, facing adverse conditions such as an acidic pH, reactive oxygen species (ROS) and nitrogen species (RNS), antimicrobial peptides, acid hydrolases, low micronutrients content, and non-physiological concentrations of heavy-metal ions [3, 4].

The concentrations of Cu^2+^, Fe^2+^, and Zn^2+^ inside phagosomes infected with *M. tuberculosis* increase between 1 and 24 h post-infection [5], until antibacterial levels of Cu^+^ and Zn^2+^ are reached [4]. In particular, high Cu^+^ concentrations generate a ROS response via oxidative stress, protein denaturation (by metal interaction with thiol groups), inactivation of enzymes by substitution of other metal cofactors, and membrane destabilization [6–8]. The intracellular concentration of heavy-metal cations is pivotal for the mycobacterial response to the oxidative burst within macrophages [6, 7]. Indeed, *M. tuberculosis* has two superoxide dismutases, SodA and SodC, that use Mn^2+^/Fe^2+^ and Cu^2+^ as cofactors, respectively [9, 10]. Other enzymes involved in the mycobacterial response to oxidative stress, such as catalase-peroxidase (KatG), alkyl hydroperoxide reductase (AhpC), and thioredoxins (TrxA and TrxB) use Fe^2+^ as a cofactor [11]. Therefore, the ion homeostasis of heavy-metal cations is essential for mycobacterial viability. The normal levels of reactive oxygen species (ROS) and nitrogen species (RNS) in macrophages are able to produce DNA and lipid damage, disrupting the activity of proteins containing Fe-S clusters, heavy-metals cations, hemes, thiols, sulfhydryl, or tyrosyl groups in phagocytosed pathogens [12, 13]. The damage mediated by ROS and RNS leads to a general inhibition of the intracellular processes associated with proton-dependent active transport, oxygen utilization, and oxidative phosphorylation [14]. In addition, bacterial pathogens to face the intraphagosomal environment produce enzymes that interfere with the synthesis of reactive species, allow the direct degradation of intermediary products, or participate in the DNA repair [13].

P-type ATPases may play an important role in mycobacterial ion homeostasis, since these usually transport cations against the concentration gradient [15]. *M. tuberculosis* has many P-type ATPases annotated as possible transporters of heavy-metal cations, suggesting that these proteins could play a relevant role in *M. tuberculosis* survival [16]. Some mycobacterial P_1B_-type ATPases have been associated with bacterial detoxification of heavy metals cations, such as Cu^+^, Zn^2+^, Co^2+^, and Ni^2+^. Specifically, CtpG, CtpV, CtpC, and CtpA are activated in human macrophages infected with *M. tuberculosis* [17]. In addition, some of these are also involved in *M. tuberculosis* virulence in animal models [18]. Indeed, CtpC and CtpV respond to an increase in the intraphagosomal concentrations of Cu^+^ and Zn^2+^ during the infection process [19].

*M. tuberculosis* possesses three putative copper transporting P-type ATPases: CtpA, CtpB, and CtpV [16]. The functional differences among these are not well understood. The *M. tuberculosis ctpA* gene is activated during the tuberculous infection of human cells [20]. On the other hand, the *ctpV* gene belongs to the *cso* “copper-sensitive operon”, which includes *csoR*, *rv0968*, *ctpV*, and *rv0970* [21] under the control of the Cu^+^-responding transcriptional regulator CsoR [22]. Interestingly, *csoR* is part of the regulon of PhoP, one global regulator of *M. tuberculosis* virulence [23]. Therefore, it has been suggested that P_1B_-type ATPases are key for controlling the intracellular content of heavy metals and delivering cofactors to extracytoplasmic enzymes [24, 25].

Interestingly, the *M. tuberculosis ctpB* gene is overexpressed under in vitro stress conditions, such as hypoxia that mimics the hostile intraphagosomal environment [26], suggesting that CtpB could be involved in tuberculous infection progress. However, the enzymatic features of CtpB and its possible role in heavy-metal cations transport across the mycobacterial plasma membrane are unknown. In this work, different functional assays were performed to evaluate the ion specificity of CtpB and its transcriptional response against stress conditions. Our findings suggest that *M. tuberculosis* CtpB is associated with Cu^+^ pumping from mycobacterial cells possibly in response to stress conditions. Furthermore, an alternative role of CtpB different from copper detoxification is discussed.

## Results

### CtpB has typical P-type ATPase copper transporting domains

**Fig. 1:**
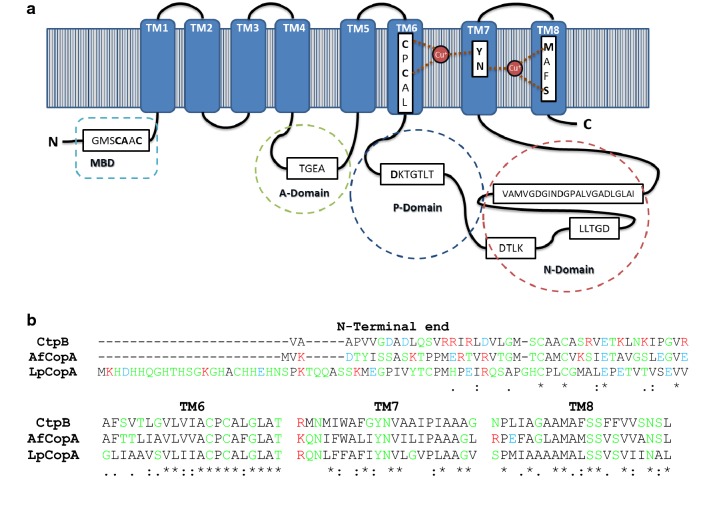
Topology, functional motifs, and domains of *M. tuberculosis* CtpB. **a** The predicted topology of CtpB shows eight TM helices (1 to 8), locating the N- and C-terminal ends within the cytoplasmic portion. The cytoplasmic domains are represented by stitched lines and the functional motifs of P_1B_-type ATPase are shown. The amino acids responsible for cation coordination within TM segments 6, 7 and 8 are highlighted in bold. **b** The TM helices were predicted using TOPCONS. The multiple alignment of the N-terminal end, TM6, TM7, and TM8 of CtpB and other bacterial characterized Cu^+^-ATPases (CopA from *Archaeoglobus fulgidus* (WP_010877980.1) and *Legionella pneumophila* (YP_095057.1)) was constructed using Clustal Omega. Some of the amino acids involved in the coordination of Cu^+^ are located within TM6, 7 and 8. The residues are classified as nonpolar (black), polar (green), acid (blue), and basic (red)

The *ctpB* gene encodes for a putative protein of 752 amino acids (77.5 kDa) [27]. In silico predictions show that CtpB belongs to the P-type ATPase family and is annotated as an acid anhydride hydrolase (EC 3.6.3.-) that catalyzes the transmembrane movement of substances. The amino acid sequence of CtpB contains eight TM segments arranged in a Type I topology [28], common to P_1B_-type ATPases (Fig. 1). The three well-known cytoplasmic domains (A: actuator, P: phosphorylation, and N: nucleotide binding) are observed in loops within TM4-5 and TM6-7 segments (Fig. 1).

Analysis of functional motifs obtained by the InterProScan server [29] also showed that CtpB is a putative copper/heavy-metal P-type ATPase transporter. The characteristic functional motifs reported by Thever and Saier [28] were identified in CtpB, with subtle differences. Specifically, CtpB showed the following functional motifs: TGEA associated with phosphatase activity; CPCAL located in the TM6 segment, providing two Cys necessary to coordinate the cation [30]; DKTGTLT that contains the phosphorylation site and is located in the largest cytoplasmic loop between TM4 and TM5 segments; DTLK and LLTGD motifs that catalyze the transfer of the phosphate group; and finally the motif known as hinge (VAMVGDGINDGPALVGADLGLAI) that provides the necessary flexibility for the conformational changes during the catalytic cycle of the enzyme [16].

Usually, the amino acids responsible for recognizing the transported cation form a binding pocket within TM6, 7 and 8, and display the adequate geometry, size, and charge for cation binding in P_1B_-type ATPases [15]. An alignment between TM6-8 segments of CtpB and the N-terminal end of the well-known Cu^+^-ATPases CopA of *A. fulgidus* and *L. pneumophila* showed that all the aligned sequences displayed the three characteristic motifs of Cu^+^-ATPases (CPC, YN, and MXXS) [31], containing six amino acids responsible for the coordination of Cu^+^ in CtpB within TM6, 7, and 8, respectively (Fig. 1b).

Some P_1B_-type ATPases have cytoplasmic metal binding domains (MBD) at the N-terminal end [32]. In this case, CtpB also showed the MBD characteristic motif GMSC(S/A)AC of P-type ATPases, where Cys could be responsible for Cu^+^ coordination. On the other hand, CtpB displayed all the residues responsible for metal coordination present in the Cu^+^-ATPase of *L. pneumophila Lp*CopA: Cys382, Cys384, and Tyr688 (binding site I), together with Asn689, Met717, and Ser721 (binding site II) [33] (Fig. 1b). Therefore, since Cu^+^ coordination is normally associated with only three coordination bonds in heavy-metal P-type ATPases [30], the presence of six residues possibly associated with metal coordination suggest that CtpB could be able to coordinate and transport at least two cations during the catalytic cycle.

### *Mycobacterium smegmatis* cells expressing *M. tuberculosis* CtpB tolerate toxic concentrations of copper

**Fig. 2:**
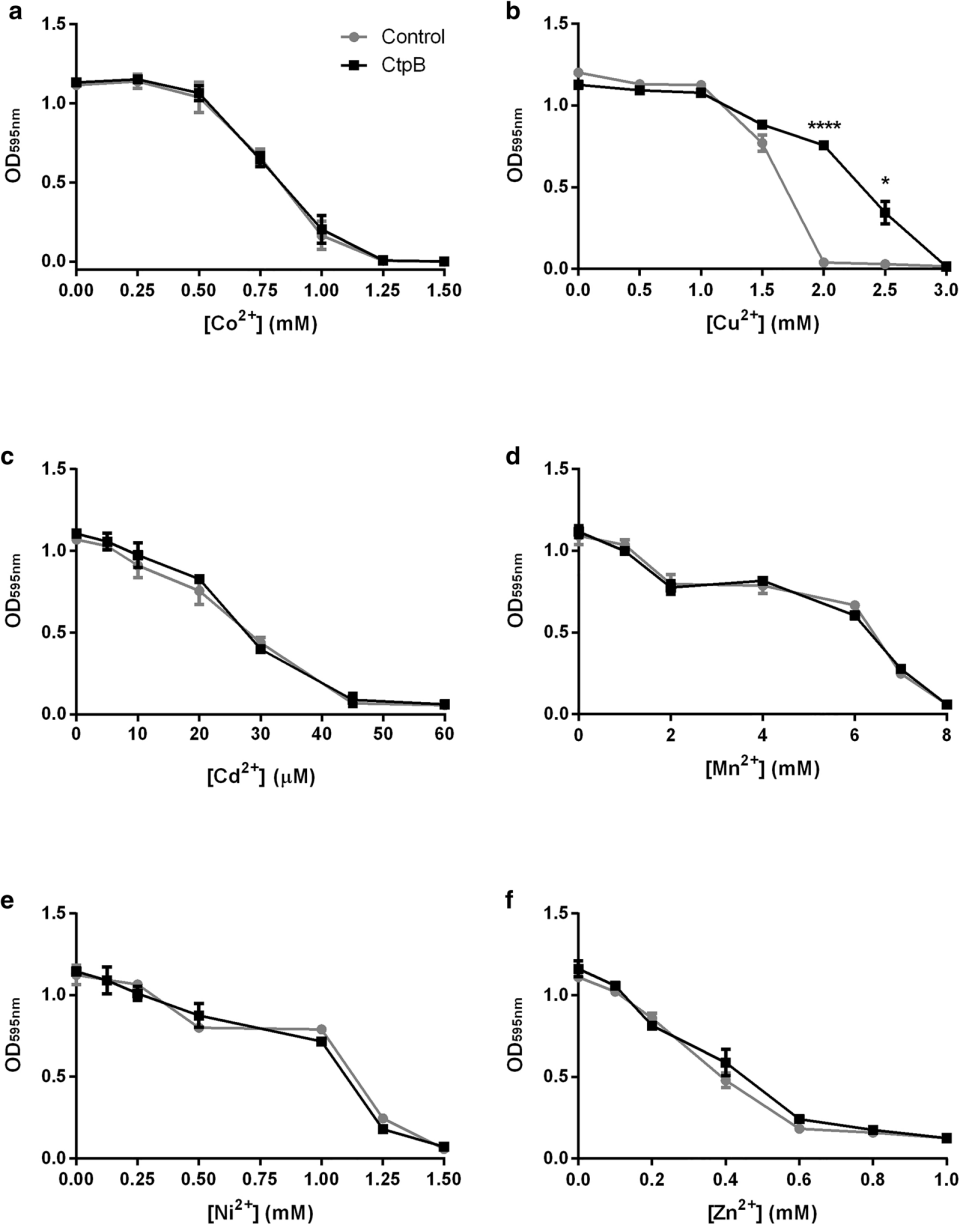
Tolerance of *M. smegmatis* cells expressing CtpB in presence of high doses of heavy-metal cations. Cultures in LB supplemented with increasing concentrations of cations: **a** Co^2+^, **b** Cd^2+^, **c** Cu^2+^, **d** Mn^2+^, **e** Ni^2+^, and **f** Zn^2+^ were incubated during 72 h at 37 °C and the OD_595_ was measured. The absorbance measured for mycobacterial cells expressing CtpB (transformed with pALT10) and control cells (transformed with pMV261) is shown in gray and black lines, respectively. The data correspond to the mean OD_595_ ± SEM from three independent experiments. Significant differences correspond to values of *P ≤ 0.05 and **** P ≤ 0.0001

We assessed the tolerance of mycobacterial cells expressing the recombinant protein in cultures supplemented with toxic concentrations of different heavy-metal cations. Since *M. smegmatis* is an adequate non-pathogenic model for the heterologous expression of *M. tuberculosis* proteins [34], *M. tuberculosis* CtpB was cloned and expressed in the plasma membrane of *M. smegmatis* mc^2^155. Nevertheless, the overexpressed *M. tuberculosis* CtpB protein in mycobacterial cells could not be detected from crude protein extracts by SDS-PAGE. Only low amounts of the CtpB protein (approximately 78 kDa) were detected in the cell membrane fraction of induced recombinant cells (*M. smegmatis* transformed with pALT10) compared to control cells transformed with the expression vector pMV261 (Additional file 1). As shown in Fig. 2, *M. smegmatis* cells expressing CtpB did not tolerate high doses of Co^2+^, Cd^2+^, Mn^2+^, Ni^2+^, and Zn^2+^. In contrast, recombinant mycobacterial cells tolerated high concentrations of Cu^2+^ ranging from 2.0 to 2.5 mM, which are usually toxic in mycobacteria. Specifically, the recombinant cells tolerated approximately twofold more Cu^2+^ concentration than control cells (*M. smegmatis* transformed with pMV261), suggesting that CtpB may be involved in copper pumping from mycobacterial cells.

### CtpB shows high affinity/low turnover for Cu^+^ in the mycobacterial plasma membrane

**Fig. 3:**
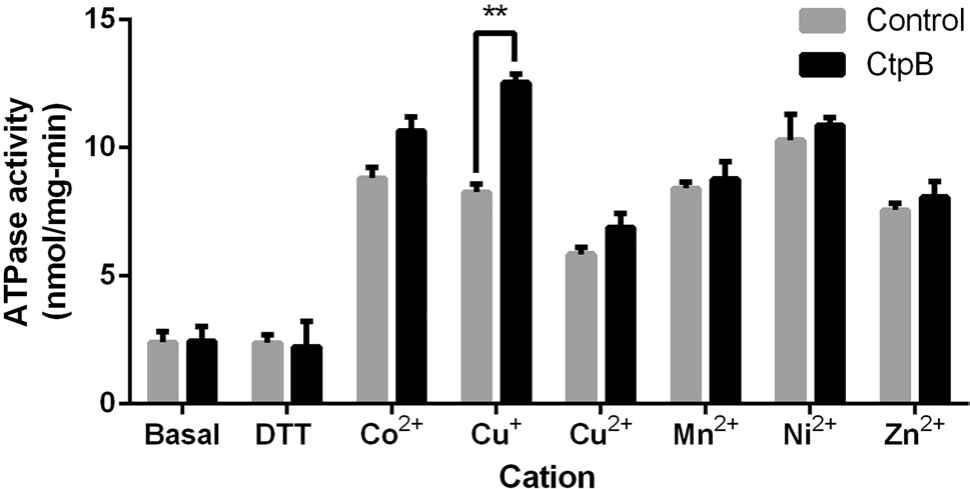
ATPase activity of CtpB in the presence of heavy metal cations. The ATPase activity was measured using plasma membranes isolated from *M. smegmatis* mc^2^155 expressing CtpB (transformed with pALT10, black bars) and control cells (*M. smegmatis* transformed with pMV261, gray bars). The values of ATPase activity were measured separately by supplementing the enzymatic reactions with Co^2+^, Cu^+^, Cu^2+^, Mn^2+^, Ni^2+^, and Zn^2+^ (at 10 μM final concentration). The data correspond to the mean ATPases activity ± SEM derived from measurements of technical triplicates. Significant differences are values of **P ≤ 0.01

The ATPase activity stimulated by different heavy-metal cations was measured in the plasma membrane of *M. smegmatis* mc^2^155 cells expressing *M. tuberculosis* CtpB. Since there is a contribution to the ATPase activity from other cell-membrane ATPases, parallel enzymatic reactions using membranes from *M. smegmatis* mc^2^155 transformed with the empty vector pMV261 were used as control. Therefore, the enzymatic activity mediated by CtpB was calculated by subtracting the ATPase activity of the control membranes from the ATPase activity of the recombinant membranes [35–37]. Using this experimental approach, it was observed that Cu^+^ was the only cation that significantly stimulated the ATPase activity of CtpB embedded in the mycobacterial plasma membrane, compared with other divalent heavy metal cations (Fig. 3). No statistically significant differences were found when the enzymatic reactions were supplemented with DTT and without Cu^+^ compared to the same enzymatic reaction without DTT. Specifically, the Cu^+^ ATPase activity obtained for CtpB recombinant membranes was 12.31 ± 0.14 nmol/mg-min.

**Fig. 4:**
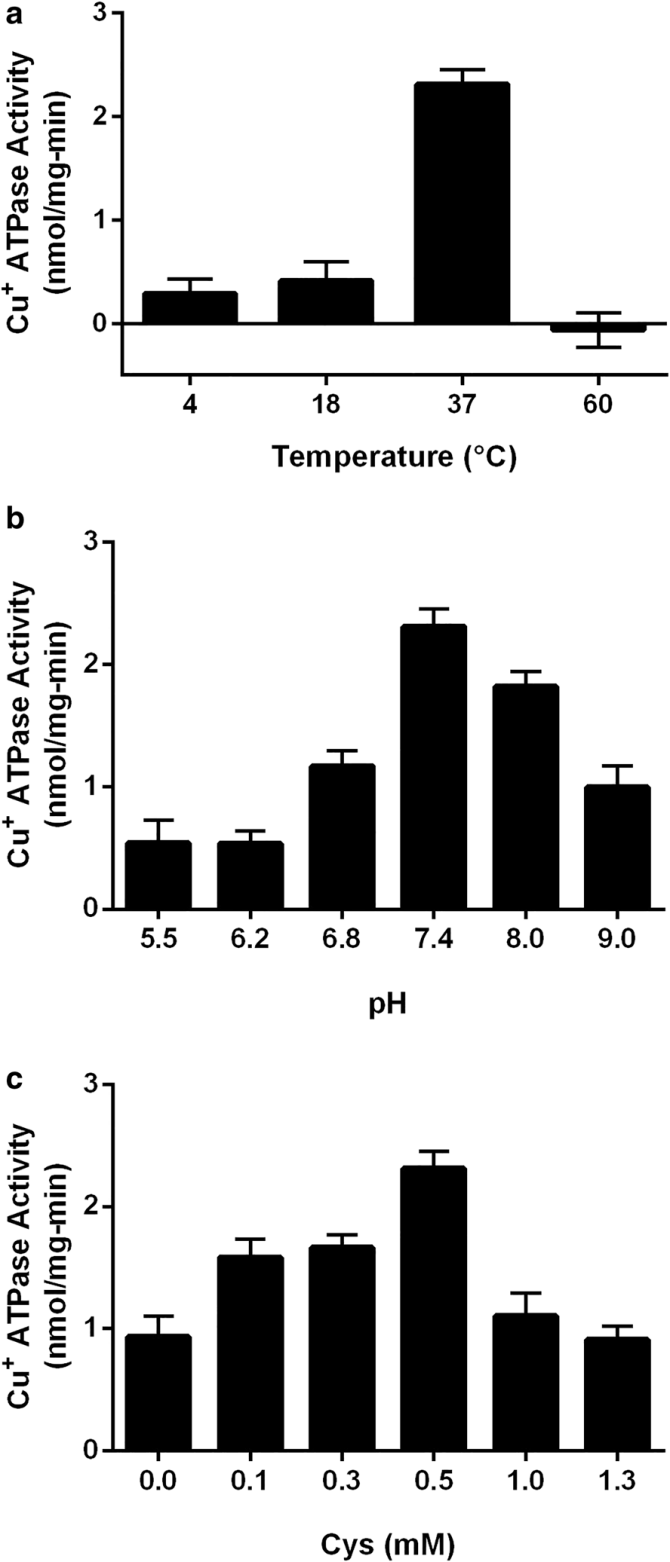
Optimal conditions for Cu^+^ ATPase activity mediated by CtpB. The Cu^+^ ATPase activity was measured using plasma membranes isolated from *M. smegmatis* mc^2^155 expressing CtpB and membranes isolated from *M. smegmatis* transformed with the expression vector pMV261. The enzymatic parameters evaluated were: **a** temperature, **b** pH, and **c** cysteine concentration. The data correspond to the mean ATPases activity ± SEM derived from measurements of technical triplicate

On the other hand, Fig. 4 shows that 37 °C is the optimal temperature among the tested temperatures for the enzymatic reaction of CtpB. This optimal temperature is similar to the average temperature of the human body, which in turn is the natural host of *M. tuberculosis*. Similarly, the observed optimal pH (7.4) is close to neutrality, as well as the observed for other mycobacterial P-type ATPases [35–37]. Finally, CtpB displayed a concentration-dependent Cu^+^ ATPase activity when the enzymatic reaction was supplemented with Cys at concentrations below 0.5 mM. In conclusion, the optimal conditions for further estimation of CtpB kinetic parameters are 0.5 mM cysteine, pH 7.4, and 37 °C.

**Fig. 5:**
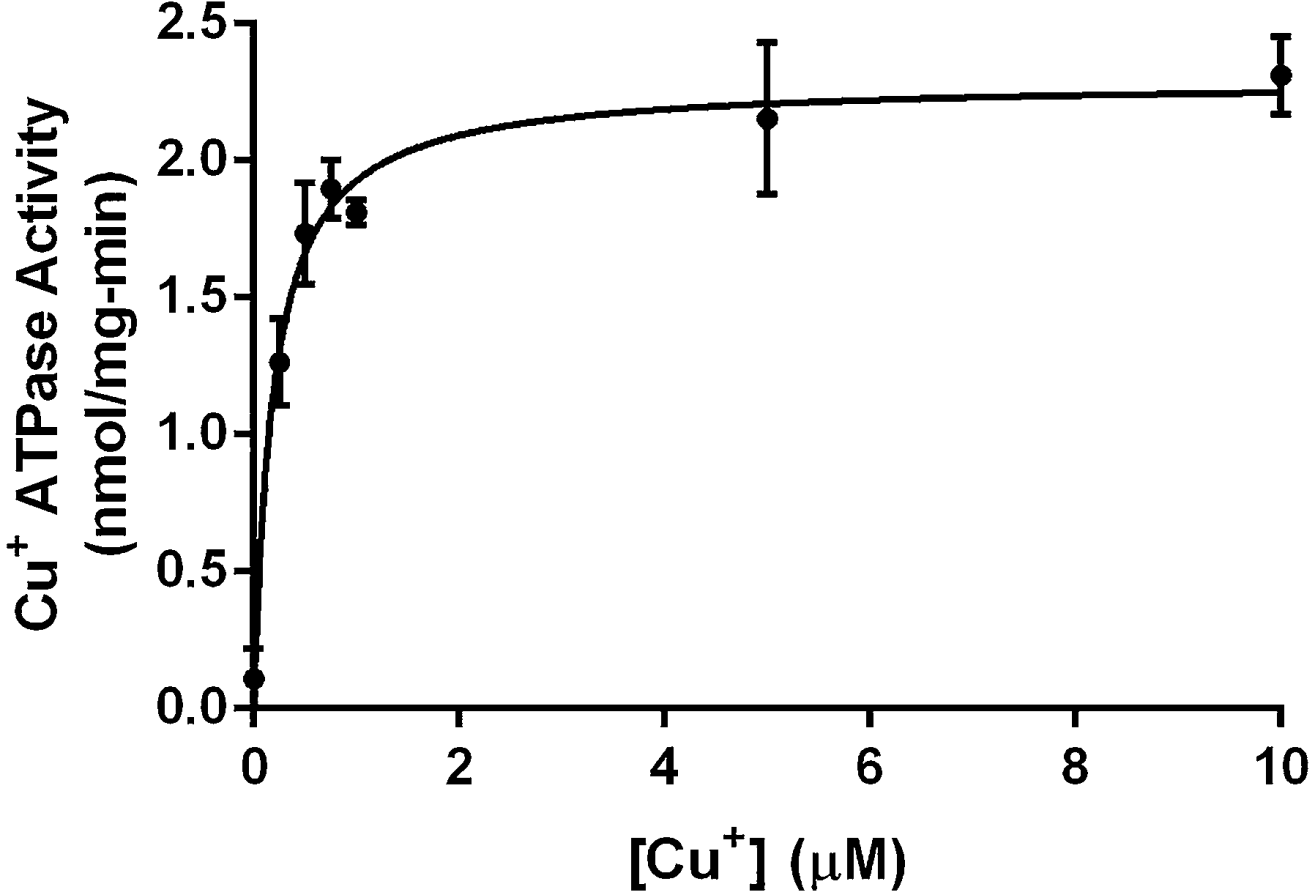
Kinetic parameters of CtpB. The Cu^+^ ATPase activity of CtpB follows a Michaelis–Menten kinetics. Cu^+^ was added at different concentrations (0.25 to 10.0 μM) and the released phosphate from the hydrolysis of ATP was quantified in the enzymatic reactions. The kinetic parameters for CtpB embedded in the plasma membrane (*K*_*M*_ 0.19 ± 0.04 μM and *V*_*max*_ 2.29 ± 0.10 nmol/mg min) were calculated using the Prism v6.0 for Windows GraphPad Software, La Jolla California USA, http://www.graphpad.com. The plotted data correspond to the average activity ± SEM from three independent experiments

The optimal conditions observed for the ATPase activity were used to estimate the kinetic parameters of CtpB. When the concentrations of Mg^2+^ and ATP are constant, CtpB catalyzes ATP hydrolysis in a Cu^+^ concentration-dependent manner, following a Michaelis–Menten kinetics (Fig. 5 and Additional file 2). CtpB embedded in the plasma membrane reached the maximal activity in vitro when reactions were supplemented with 5 μM Cu^+^. In addition, CtpB displayed a *V*_*max*_ of 2.29 ± 0.10 nmol/mg-min and *K*_*M*_ of 0.19 ± 0.04 µM Cu^+^.

### *ctpB* disruption does not alter the growth rate and copper accumulation of *M. tuberculosis* cells

**Fig. 6:**
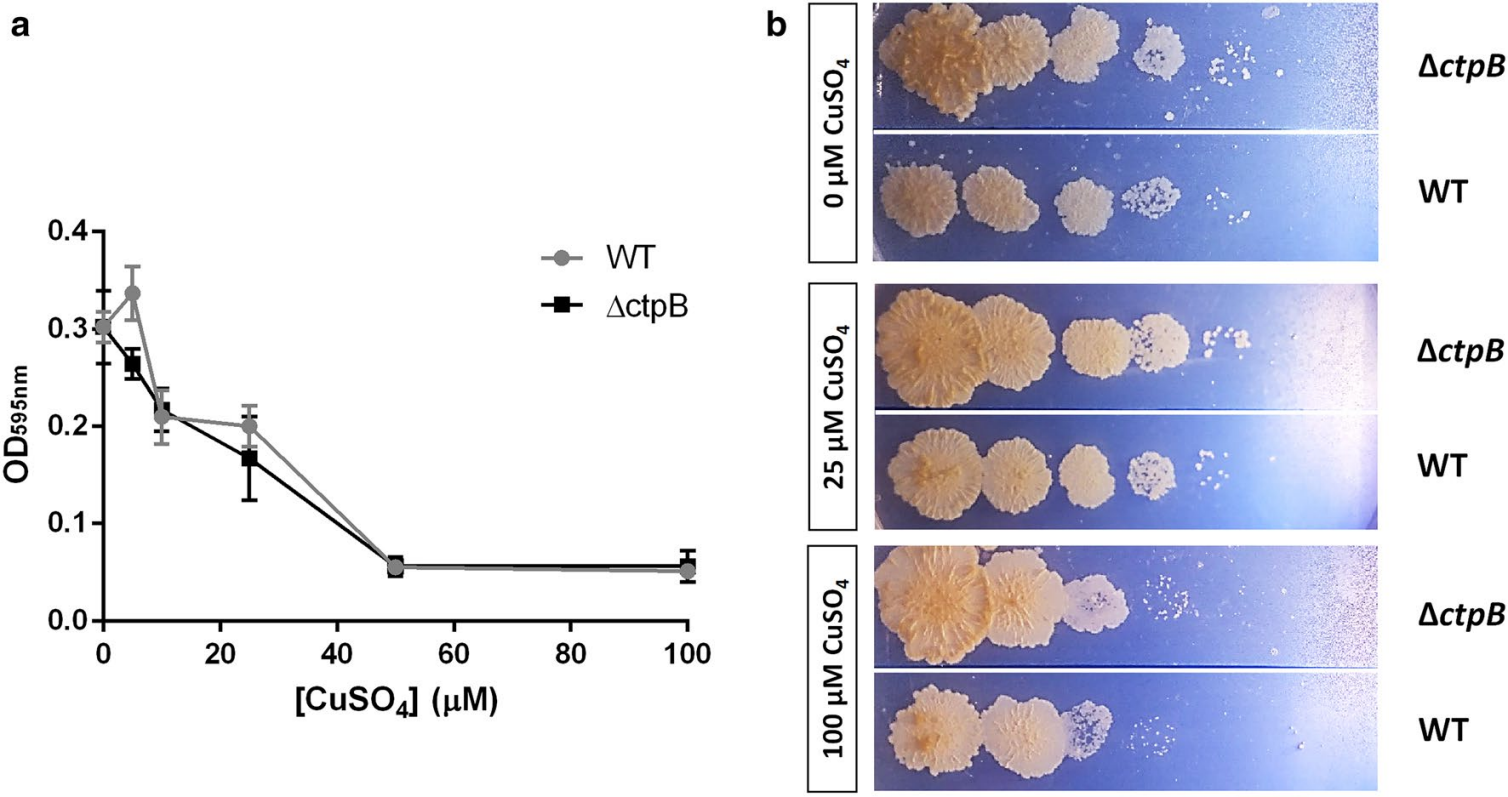
Effect of Cu^2+^ in the growth rate of *ctpB*-disrupted *M. tuberculosis* cells. **a** The growth rate of *M. tuberculosis* Δ*ctpB* (black) and *M. tuberculosis* H37Ra wild-type (gray) cells in modified Sauton medium supplemented with high doses of Cu^2+^ was measured at OD_595_. The data are shown as the mean ± SEM from three independent experiments. **b** Serial dilutions (tenfold) of *M. tuberculosis* cells (wild type and *ΔctpB* cells) previously grown until mid-log phase were seeded and grown on 7H11-OADC plates supplemented with 0.25 and 100 μM CuSO_4_

**Fig. 7:**
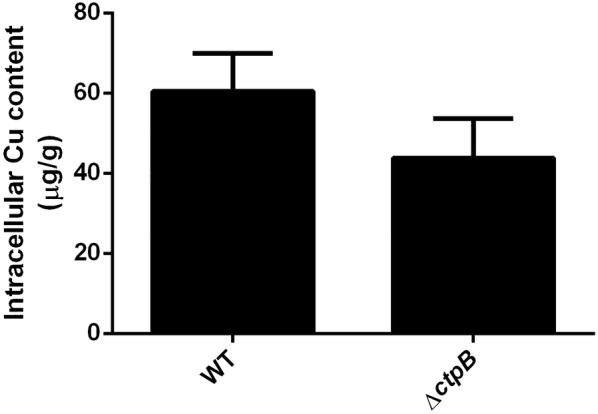
Copper accumulation in whole *M. tuberculosis ΔctpB* cells. The content of intracellular copper in *M. tuberculosis* wild-type and Δ*ctpB* cells previously treated with 50 μM of CuSO_4_ was assessed by AAS-GF. The values of metal accumulation are shown as μg of Cu/g of dry pellet. The data are shown as the mean ± SEM from three independent experiments

*ctpB* disruption obtained by recombineering in *M. tuberculosis* cells (*ΔctpB*) was corroborated by PCR (Additional file 3). Initially, we conducted assays of mycobacterial tolerance to toxic concentrations of divalent metals to evaluate if CtpB is involved in copper detoxification of mycobacterial cells, which would result in the susceptibility of *M. tuberculosis ∆ctpB* cells to toxic doses of copper. Notably, the growth rate of *∆ctpB* cells cultured in broth or solid medium under high doses of Cu^2+^ did not show statistically significant differences compared with wild type cells cultured under the same experimental conditions (Fig. 6). The possible role of CtpB in copper detoxification was also evaluated by comparing copper accumulation in wild-type and mutant cells. Similarly, the experiments of atomic absorption showed that there were no significant differences in copper accumulation between *M. tuberculosis* wild-type and *∆ctpB* cells cultured under high doses of copper (Fig. 7). Therefore, if CtpB were responsible for copper detoxification, gene disruption should reduce cation efflux and copper should accumulate inside the *∆ctpB* cells. Conversely, both strains showed similar copper accumulation, corroborating that CtpB is not directly associated with copper detoxification in mycobacterial cells. Therefore, the assays of mycobacterial tolerance to high doses of cations and the metal accumulation assays strongly suggest that CtpB is not directly associated with copper detoxification, whereas CtpB could play an alternative role in the copper transport system mediated by P-type ATPases across the mycobacterial plasma membrane.

### CtpB is associated with the response to stress conditions in *M. tuberculosis* cells

**Fig. 8:**
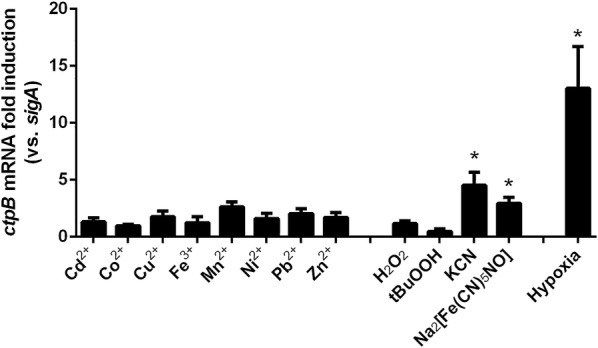
Transcriptional analysis of CtpB under stress conditions. Relative transcription of *ctpB* from cultures separately supplemented with different concentrations of heavy-metal cations (Cd^2+^, Co^2+^, Cu^2+^, Fe^2+^, Mn^2+^, Ni^2+^, Pb^2+^, and Zn^2+^), hypoxia, and 1 mM of different redox stress agents (H_2_O_2_, tert butyl hydroperoxide, KCN, and sodium nitroprusside). *sigA* was used as the normalizer gene. The data were plotted as the average values ± SEM from three independent experiments. The differences (*P < 0.05) were obtained by comparing the values in the presence and absence of the stress conditions

In order to gain further insight into the possible role of CtpB during mycobacterial copper transport, a transcriptional analysis of *ctpB* was assessed in vitro under experimental conditions mimicking the intraphagosomal environment: the presence of high doses of heavy-metal cations, hypoxia, and redox stress (Fig. 8). We observed that *ctpB* transcription was activated 4.53 ± 1.12 and 2.94 ± 0.52-fold by redox stressors KCN and sodium nitroprusside, respectively. Similarly, hypoxia strongly induced *ctpB* transcription (13.02 ± 3.69-fold) compared with cells cultured under complete aeration. Conversely, *ctpB* transcription was not significantly induced by the presence of toxic doses of heavy-metal cations.

## Discussion

Bacterial tolerance against toxic concentrations of metal cations mediated by P-type ATPases has been previously reported in mycobacteria. For example, recombinant *M. smegmatis* cells overexpressing the putative *M. tuberculosis* cadmium transporter CtpG tolerate high doses of Cd^2+^ compared with non-recombinant mycobacterial cells [36]. Similarly, *M. smegmatis* homologously expressing the putative alkali/alkaline earth cation P-type ATPase transporter Pma1 tolerates toxic concentrations of Na^+^, K^+^, and Na^+^/K^+^ ions [37]. Regarding P-type ATPases associated with copper transport, we previously reported that mycobacterial cells expressing the putative *M. tuberculosis* CtpA, a P-type ATPase copper transporter, grow approximately twofold more than non-recombinant cells in the presence of toxic copper concentrations (from 1 to 4 mM), suggesting a possible role of CtpA in copper detoxification in mycobacteria [35]. Therefore, CtpB is likely associated to Cu^+^ pumping through the mycobacterial plasma membrane, based on the typical P-type ATPase Cu^+^ transporting domains shown by the bioinformatics analysis of CtpB. In this sense, the fact that *M. smegmatis* cells expressing CtpB tolerate toxic levels of Cu^2+^, and that the ATPase activity mediated by this transporter is stimulated by Cu^+^ but not Cu^2+^, means that the toxic divalent cation (Cu^2+^) enters the mycobacteria, where it is reduced to the monovalent form (Cu^+^) by the reducing environment of the mycobacterial cytoplasm [38]. Thus, Cu^+^ can be extruded to the external environment by CtpB, like previously observed for *M. tuberculosis* CtpA [35]. In addition, it is known that Cys chelates copper, which is comparable with the function of Cu^+^-chaperones that deliver the cation to the ATPases [39]. This suggests that CtpB could prefer the binding of chelated Cu^+^, which is common in the mycobacterial intracellular environment, where the thiol group of proteins binds Cu^+^. As a result, the presence of intracellular free Cu^+^ becomes practically undetectable [40]. On the other hand, the *V*_*max*_ (2.29 ± 0.10 nmol/mg-min) and *K*_*M*_ (0.19 ± 0.04 µM Cu^+^) of CtpB are lower than those observed for other P_1B_-type ATPases, such as *E. coli* CopA (*V*_*max*_ of = 27.3 ± 4.5 nmol/mg-min) [41] or ATP7A (*K*_*M*_= 0.6 ± 0.04 µM) [42], respectively. This means that CtpB is a less-efficient copper transporter with more affinity and a lower turnover of Cu^+^ compared to these plasma membrane transporters. However, we cannot rule out that the kinetic parameters of CtpB were measured using plasma membrane fractions, in which other proteins could affect the specific activity of the enzyme and the observed values for *V*_*max*_.

It was previously reported that *M. tuberculosis* CtpV mutant cells do not tolerate toxic doses of copper [6], indicating that CtpV could be the P-type ATPase directly associated with copper detoxification in *M. tuberculosis.* This finding suggests that the other putative copper P-type ATPase transporters encoded in the *M. tuberculosis* genome, such as CtpA and CtpB, could play alternative roles during copper transport across the mycobacterial plasma membrane. This is the case of Cu^+^-ATPases in *P. aeruginosa* and *Sinorhizobium meliloti*. Similarly, these bacterial species contain more than two genes coding for Cu^+^-ATPases in the genome, as with the *M. tuberculosis* genome. When Cu^+^-ATPases are encoded by at least two genes in bacteria, one of the encoded enzymes performs an alternative function to metal detoxification, such as metalation of periplasmic and membrane proteins [24]. Indeed, *P. aeruginosa* contains two paralogous Cu^+^-ATPases: CopA1 (*V*_*max*_ 63.1 ± 1.5 nmol/mg min and *K*_*M*_ 152.6 ± 7.9 μM Cu^+^), which mediates copper detoxification, and CopA2 (*V*_*max*_ 6.7 ± 0.4 nmol/mg min and *K*_*M*_ 20.7 ± 3.7 μM Cu^+^) that is involved in cytochrome c oxidase metalation [43]. Therefore, if the kinetic parameters obtained for CtpB are closer to CopA2 than CopA1, it could be presumed that CtpB may play a protein metalation role, like CopA2. In agreement, knock-out of genes coding for Cu^+^-ATPases responsible for protein metalation in *P. aeruginosa* and *S. meliloti* does not result in a copper-sensitive phenotype [43, 44]. This finding is similar to our observations for *M. tuberculosis ∆ctpB* cells and enforces the possibility that CtpB is associated with enzyme metalation. Furthermore, it agrees with our results of copper accumulation and mycobacterial tolerance to high doses of cation displayed by *M. tuberculosis* ∆*ctpB* mutant cells.

On the other hand, hypoxia and redox stress seem to be correlated in mycobacterial cells. Specifically, it is known that sodium nitroprusside produces nitric oxide (NO), which is able to induce the expression of genes related to hypoxia and affect the aerobic respiration of mycobacteria [45]. NO and CN^−^ inhibit the CtaD subunit of the cytochrome c oxidase, which belongs to the chain of electron transport in mycobacteria. When cytochrome c oxidase is inhibited, mycobacteria respond using the cytochrome bd quinol oxidase, which has a greater affinity for oxygen compared with the cytochrome c oxidase [46]. Therefore, the transcriptional behavior of *ctpB* described above suggests that the oxidative stressors NO and CN^−^ activate *ctpB* transcription, possibly facilitating the switching from aerobic respiration to hypoxia. Unlike hypoxia and redox stress, the transcriptional analysis also showed that *ctpB* was not significantly induced by Cu^2+^. This finding reinforces the idea that CtpB is associated with a transport function different from copper detoxification. It is possible that CtpB translocates Cu^+^ from the cytoplasm to the well-known cuproenzymes in the periplasm, such as, superoxide dismutase (SodC), cytochrome oxidase, and multicopper oxidase (MmcO), which use copper as cofactor [10, 24, 47]. In this case, CtpB would be performing an enzyme metalation function, which in turn is involved in the response to hypoxia and redox stress, as suggested by the transcriptional analysis of *ctpB* in *M. tuberculosis* cells, along with the growth rate and copper accumulation of *M. tuberculosis* ∆*ctpB* cells described above.

## Conclusions

Our data indicate that CtpB is a copper (I) transporting P-type ATPase of *M. tuberculosis*, which induces mycobacterial tolerance to toxic concentrations of Cu^2+^. However, CtpB shows a metal preference for Cu^+^ in a metal concentration-dependent manner suggesting that Cu^2+^ enters the mycobacteria, where it is reduced to Cu^+^. On the other hand, the Cu^+^ ATPase activity of CtpB in the plasma membrane shows features of high affinity/slow turnover ATPases similar to Cu^+^ transporting ATPases involved in protein metalation. Finally, since the *ctpB* gene is transcriptionally activated under culture conditions that mimicked the stress conditions similar to the intraphagosomal environment, but not under high doses of copper, the overall results suggest that *M. tuberculosis* CtpB is associated with copper (I) pumping from mycobacterial cells possibly playing a role different from copper detoxification.

## Materials and methods

### Bioinformatics analysis

The amino acid sequence of CtpB was obtained from Turberculist [27]. The protein topology was predicted with TOPCONS [48], and functional motifs were located as shown by Novoa-Aponte [16]. Clustal OMEGA [49] was used to align the amino acid sequences of *M. tuberculosis* CtpB with well-characterized bacterial Cu^+^-ATPases, including CopA from *Archaeoglobus fulgidus* (WP_010877980.1) and *Legionella pneumophila* (YP_095057.1). The transmembrane (TM) positions were predicted with TOPCONS server [48].

### Bacterial strains and growth conditions

*M. tuberculosis* H37Ra was grown in Middlebrook 7H9 or 7H11 supplemented with 10% OADC (50 μg/mL oleic acid, 0.5% Bovine albumin Fraction V, 0.2% dextrose, and 0.004% catalase), or Sauton medium supplemented with 0.2% glucose, 30 μM Fe_2_(SO_4_)_3_, and 0.05% Tween-80. *M. smegmatis* mc^2^155 cells were grown in LB or Sauton medium supplemented as described above. Mycobacteria were grown until an OD_595nm_ = 0.4 for electroporation experiments or OD_600nm_ = 0.6 for plasma membrane extraction. *Escherichia coli* TOP10 and HB101 host cells were cultured in LB and used for plasmid constructions. When needed, 7H9 or 7H11 were supplemented with 10% OADC, 0.05% Tween 80, 20 μg/mL kanamycin (Km), and 50 μg/mL hygromycin (Hyg). Likewise, LB was supplemented with 50 μg/mL Km, 100 μg/mL Hyg, 100 μg/mL ampicillin (Amp), 0.5 mM isopropyl-β-d-thiogalactopyranoside (IPTG), and 80 μg/mL 5-bromo-4-chloro-3-indolyl-β-d-galactopyranoside (X-Gal).

### Cloning and expression of *M. tuberculosis* CtpB

The *ctpB* gene was amplified by PCR from the genomic DNA of *M. tuberculosis* H37Rv using the pair of primers pMV-ctpB-dir (5′-TTTTGAATTCGTGGCGGCTCCAGTTGTGG-3′)/pMV-ctpB-rev (5′-TTTTAAGCTTCCTGGATCTGACCCCGACC-3′). The resulting amplimer was cloned in the pGEM-T easy vector (Promega, WI, USA) to obtain the pALT9 recombinant plasmid. The *ctpB* gen was subcloned in the shuttle vector pMV261 [50] using the restriction sites *Hind*III–*Eco*RI, generating the pALT10 plasmid. The integrity of pALT10 was confirmed by automated sequencing. pALT10 and pMV261 were separately transformed in *M. smegmatis* mc^2^155 cells by electroporation and, finally, expression of the recombinant protein was induced by incubation of mycobacterial cells at 45 °C for 3 h [37]. The induction of CtpB expression was assessed in cells used to isolate plasma membrane fractions for the ATPase activity assays, including the optimal enzymatic conditions and kinetic parameters of CtpB.

### Construction of a *M. tuberculosis ctpB* mutant

A *M. tuberculosis* H37Ra *ctpB* mutant was obtained by recombineering [51]. For the gene mutation, an allelic exchange substrate (AES) was constructed by flanking a Hyg^R^ cassette with genomic fragments from the upstream and downstream genomic regions of the *ctpB* gene. Specifically, a 500 bp genomic fragment downstream of *ctpB* was amplified by PCR using the pair of primers Down-ctpB-dir (5′-TTTTAAGCTTATCGCCGGTGCCGCCATGG-3′)/pMV-ctpB-rev (5′-TTTTCTCGAGTCTGCCGTGGTTGTCGAGC-3′) and cloned in the pGEM-T easy vector to obtain the pALT13 plasmid. This downstream *ctpB* amplimer was then subcloned into the *Hind*III and *Xho*I sites of the pYUB584 vector to obtain the pALT15 plasmid. The upstream region of *ctpB* was amplified using the pair of primers Up-ctpB-dir (5′-TTTTCCTAGGGCGGGTGTACTGGGTGTCG-3′)/Up-ctpB-rev (5′-TTTTTCTAGATCGACGCGGCTGGCACAGG-3′) and cloned into the *Avr*II and *Xba*I sites of pALT15, obtaining the pALT16 plasmid. Finally, the *ctpB* AES (2987 pb fragment) was isolated from pALT16 by double digestion with *Xho*I and *Avr*II. A recombineering strain was separately obtained by electroporation of *M. tuberculosis* H37Ra cells with the pJV53 vector [51]. The recombineering strain was grown in 7H9-OADC-Km-Tween 80 at 37 °C until an OD_595nm_ = 0.6, then supplemented with 1.5% glycine and 0.2% acetamide, and incubated at 37 °C, 80 rpm during 18 h. Subsequently, 200 ng of *ctpB* AES were electroporated in the resulting mycobacterial cells. Finally, mutant cells (Δ*ctpB)* were selected by hygromycin resistance on 7H11-OADC-Km-Hyg plates.

### Mycobacterial tolerance to different concentration of divalent cations

The tolerance of mycobacterial cells to divalent cations was assessed under high doses of the ions that could be potentially transported by CtpB [35]. Briefly, 200 µL of mycobacterial cultures of *M. smegmatis* mc^2^155 separately transformed with pALT10 or pMV261 (control cells) were cultured in LB supplemented with 0.05% Tween-80, 25 mg/mL Km, and different concentrations of heavy metal salts, with incubation at 37 °C and 80 rpm for 72 h. Bacterial growth was measured at OD_595nm_ using the iMark ™ Microplate Absorbance Reader plate reader (Bio-Rad, USA), until an OD_595nm_ = 0.05. The tested concentrations of salts were CdCl_2_ (5 to 60 μM), CoCl_2_ (0.25 to 1.5 mM), CuSO_4_ (0.5 to 3 mM), MnSO_4_ (1 to 8 mM), NiSO_4_ (0.125 to1.5 mM), and ZnSO_4_ (0.1 to 1 mM). Each concentration of cation and strain were tested by triplicate.

The copper sensitivity of the mutant strain (*M. tuberculosis* H37Ra ∆*ctpB*) was assessed by growing the mutant cells under different CuSO_4_ concentrations (5 to 100 μM) using the wild type strain as control cells. Mutant cells were grown in 3 mL of Sauton (initial OD_595nm_ = 0.05) supplemented with 0.2% glucose, 30 μM Fe_2_(SO_4_)_3_, 0.05% Tween-80, and different concentrations of CuSO_4_. Cultures were incubated at 37 °C and 80 rpm for 3 weeks and the bacterial growth was estimated by measuring the OD_595nm_. The absorbance values were corrected by subtracting the absorbance of the negative growth control (samples without bacteria). Each copper concentration was tested in three independent experiments. An assay of mycobacterial tolerance to cations was also carried out on solid 7H11-OADC medium supplemented with CuSO_4_ (0.25 and 100 μM). Briefly, mycobacterial cultures were grown until exponential phase (OD_595nm_ = 0.5) and serially diluted in modified Sauton medium. Subsequently, 5 μL of each dilution were spotted on the agar plates. Plates were incubated at 37 ℃ for 3 weeks. This assay was done in three independent experiments.

### ATPase activity of the plasma membrane

Plasma membranes were isolated from cultures (1 L, OD_595nm_ = 0.6) of *M. smegmatis* mc^2^155 cells separately transformed with pALT10 or pMV261, as previously described by León-Torres [35], with modifications. Briefly, mycobacterial cells expressing CtpB were collected by centrifugation and washed twice with washing buffer (10 mM MOPS, 250 mM sucrose, pH 7.4). The obtained pellets were then resuspended in 2 mL of lysis buffer (10 mM MOPS–KOH, 0.3 mM EDTA, 1 mM PMSF, and 1 mg/mL lysozyme, pH 7.4) and incubated at room temperature for 30 min. Cells were lysed with a Mini Beadbeater-16 (Biospec, USA) by 4 pulses of 2 min each. The membrane fraction was then obtained by differential centrifugation at 4 ℃: first at 25,000*g* during 30 min, followed by centrifugation of the obtained supernatant at 10,000*g* during 90 min. The pellet, that corresponds to the plasma membrane fraction, was resuspended in resuspension buffer (10 mM MOPS–KOH, 250 mM sucrose, and 1 mM PMSF, pH 7.4). The membrane protein was quantified by the Bradford-Zor-Selinger method [52] and subsequently used for the enzymatic assays.

The ATPase activity stimulated by heavy-metal cations was estimated using plasma membranes isolated from *M. smegmatis* mc^2^155 cells transformed with pALT10 or pMV261 (control cells) [35]. The enzymatic reactions (50 μL) were performed in the reaction buffer (40 mM MOPS–TRIS, 3 mM MgCl_2_, 5 mM NaN_3_, 0.25 mM Na_2_MoO_4_, and 0.5 mM Cys, pH 7.4) using 20 μg of plasma membrane protein, and supplemented with 10 μM (final concentration) of the tested heavy metal cation (CoCl_2_, CuSO_4_ supplemented with 2.5 mM DTT, CuSO_4_, MnSO_4_, NiSO_4_, and ZnSO_4_). The enzymatic reactions were initiated by the addition of 3 mM Na_2_ATP, followed by incubation at 37 °C for 30 min. The ATPase activity was stopped by adding 100 μL of stop solution (3% ascorbic acid, 0.5% ammonium molybdate, and 3% SDS in 1.0 M HCl) and 150 μL of 3.5% bismuth citrate and 3.5% sodium citrate in 2.0 M HCl. Subsequently, the released inorganic phosphate (Pi) was quantified [53]. The difference between the total ATPase activity and the activity obtained from samples supplemented with no cations (basal activity) was considered as the ATPase activity stimulated by the tested cations. Enzymatic reactions supplemented with DTT and without Cu^+^ were also performed as controls of possible DTT interference in the enzymatic reaction. The ATPase activity of samples was performed by triplicate.

### Kinetic parameters of CtpB

The ATPase activity associated with CtpB was calculated as the difference between the activity of the membrane from recombinant cells (transformed with pALT10) and the activity of membrane isolated from the cells transformed with the empty vector pMV261 (control). Since we noticed that values of the enzymatic activity could vary depending on the plasma membrane isolate and it was lost over time, the same plasma membrane preparation was used to estimate the optimal enzymatic conditions and the kinetic parameters. To obtain the optimal conditions for CtpB activity, the enzymatic reactions were incubated at 37 ℃ during 30 min with the following conditions: 20 μg of plasma membrane protein, 40 mM MOPS–TRIS (pH 7.4), 3 mM MgCl_2_, 10 μM CuSO_4_, 5 mM NaN_3_, 0.25 mM Na_2_MoO_4_, 0.5 mM Cys, 2.5 mM DTT, and 3 mM Na_2_ATP. To establish the optimum pH for the enzymatic reaction, samples were tested from pH 5.5 to 9.0, using MES–KOH for pH 5.5 and 6.2, MOPS–KOH for pH 6.8 and 7.4, and Tris–HCl for pH 8 and 9. Similarly, the optimum temperature was tested between 4 and 60 °C, and the optimal concentration of Cys was evaluated by testing concentrations from 0.1 to 10 mM. After that, enzymatic kinetics were performed under the optimal reaction conditions and varying the CuSO_4_ concentration from 0.25 to 10 μM. The values of *K*_*M*_ and *V*_*max*_ were calculated using the nonlinear least squares regression for Michaelis–Menten enzyme kinetics using Prism 6 version 6.0 h for Windows 10, GraphPad Software, La Jolla California USA, http://www.graphpad.com.

### Copper accumulation assay

Assays of copper accumulation in mycobacteria were performed using the *M. tuberculosis* H37Ra ∆*ctpB* and wild-type strains, following the procedure reported by Raimunda [54], with some modifications. Briefly, 100 mL of mycobacterial culture was grown in 7H9-OADC-Tween 80 at 37 °C until an OD_595_ = 0.5. The cultured cells were then collected by centrifugation, washed thrice with one volume of modified Sauton medium, resuspended in 30 mL of the same medium, and aliquoted in samples of 5 mL. Two samples were processed in parallel, one of them was supplemented with 50 μM CuSO_4_ and the other without a cation. Samples were then incubated at 37 °C and 80 rpm for 3 h. The cells were collected by centrifugation and washed 4 times with one volume of 150 mM NaCl. The pellet was then dried at 37 °C for 24 h and weighed. The cells were mineralized with 500 μL of HNO_3_ (ultrapure quality, Merck) at 80 °C for 1 h and at room temperature overnight. Subsequently, 20 μL of 30% H_2_O_2_ were added to the samples. Finally, the copper content in the samples was determined by graphite furnace atomic absorption spectroscopy (GF-AAS) by High-Resolution Continuum Source Electrothermal Atomic Absorption Spectrometry in a contrAA^®^ 700 D (Analytikjena, Germany) equipment, following the manufacturer’s instructions. Separately, seven copper standards (4, 10, 20, 40, 60, 80, and 100 µg/kg) were prepared from a copper stock for GF-AAS of 1000 mg/kg (Acros Organics) in 0.5% HNO_3_. Samples were mixed with 5 μL of modifier (0.1% Pb(NO_3_)_2_ and 0.05% Mg(NO_3_)_2_) and then quantified for copper content at 324.754 nm using pyrolysis and atomization temperatures of 1100 and 1500 °C, respectively. Metal accumulation is reported as μg of accumulated Cu^+^/g dry cell pellet (μg Cu^+^/g); the value obtained from the control sample (without cation addition) was subtracted. The assay was performed in three independent experiments. Statistical significance was determined using the Holm-Sidek method (alpha 5.000%), using Prism 6 version 6.0 h for Windows 10, GraphPad Software, La Jolla California USA, http://www.graphpad.com.

### Transcriptional analysis of *ctpB* by qPCR

The transcriptional behavior of *ctpB* was analyzed from cells cultured under the presence of toxic concentrations of heavy-metal cations, ROS, RNS, and hypoxia. *M. tuberculosis* H37Ra cells grown until exponential phase (OD_595_ = 0.6) were washed and resuspended in Sauton medium. The cells were then intoxicated supplementing the cultures separately with CdCl_2_ (10, 25, and 40 μM), CoCl_2_ (0.45, 2, and 3.5 mM), CuSO_4_ (20, 50, and 500 μM), Fe_2_(SO_4_)_3_ (0.2, 0.41, 0.45, and 2 mM), MnSO_4_ (0.2, 0.4, 1.0, and 15 mM), NiSO_4_ (0.6, 1, and 2.5 mM), Pb(NO_3_)_2_ (0.5 mM) or ZnSO_4_ (40, 200, and 500 μM). In the case of redox stressors, cells were exposed to 1 mM of H_2_O_2_, tert-butyl-hydroperoxide (tBuOOH), KCN, or sodium nitroprusside (Na_2_[Fe(CN)_5_NO]). Then, the cells were incubated for 3 h at 37 °C and 80 rpm, harvested and used for RNA isolation. Similarly, the cultures were also subjected to a non-replicative persistence 2 (NRP-2) or anaerobic stage, as described by Wayne and Hayes [55], and compared with standard cultures. In this case, a parallel culture supplemented with methylene-blue (1.5 µg/mL) was used as an indicator of oxygen depletion.

RNA from *M. tuberculosis* cultures was isolated using the TRIzol method (Invitrogen, USA) [56]. Total RNA was dissolved in DEPC-treated H_2_O and stored at − 70 °C. cDNA from all samples was prepared using 2 µg of RNA and the *RevertAid First Strand cDNA Synthesis* kit (Thermo Scientific, USA). *ctpB* transcription was quantified by qRT-PCR using the specific primers (5′-ACATCCATGGAAACGCTGAT-3′/5′-CAAGAACGAAGACGGTCACA-3′) and normalized to the mean value of *sigA* gene expression (5′-CCTACGCTACGTGGTGGATT-3′/5′-TGGATTTCCAGCACCTTCTCC-3′). Fluorescence was quantified using the *iQ™ SYBR*^*®*^*Green Supermix* kit (Bio-Rad, USA) on the CFX-96 thermocycler (Bio-Rad, USA). To validate the qRT-PCR assays and determine their efficiency, serial dilutions of genomic DNA of *M. tuberculosis* H37Ra were tested. Quantification was performed four times for each condition, and results were analyzed using a calibration curve in which the regression and efficiency values were close to 1 and 2, respectively. In all cases, the relative quantification was analyzed using the Pfaffl method [57]. Reactions were performed in triplicate from two independent experiments, and differences between the experimental data were considered significant at *P* < 0.05.

## Supplementary information

**Additional file 1.*** M. tuberculosis* CtpB expression in* M. smegmatis* plasma membrane. SDS-PAGE analysis of CtpB in the mycobacterial plasma membrane. 12% SDS-PAGE analysis was performed with the membrane fraction of the different cells: pALT10, *M. smegmatis* expressing CtpB; pMV261, *M. smegmatis* transformed with the expression vector pMV261 (control without CtpB). The arrow shows a membrane protein of approximately 78.7 kDa corresponding to the molecular weight of the CtpB recombinant protein.

**Additional file 2.** Enzymatic reaction progress of CtpB. We monitored the Cu^+^ ATPase reaction progress by measuring the released Pi (nmol) at different enzymatic reaction times. The assay was performed using 10.0 μM Cu^+^ and enzymatic reactions times between 0 and 60 min. The plotted data correspond to the average released Pi (nmol) ± SEM from three independent experiments.

**Additional file 3.** Disruption of *ctpB* in *M. tuberculosis* H37Ra cells. a) Schematic representation of the *M. tuberculosis* H37Ra *ctpB g*enomic region, indicating the primers used to verify the mutant genotype (negative control). **b)** Schematic representation of the *M. tuberculosis* H37Ra genome, where *ctpB* is interrupted by a Hyg cassette (positive control); the primers used for PCR amplification are shown. **c)** PCR of the genomic DNA isolated from three possible *M. tuberculosis* H37Ra ΔctpB colonies using different primer combinations. Genomic DNA from the wild-type strain (WT) and pALT16 plasmid (AES) were used as controls.

## Data Availability

All the data analyzed during this study are included in this article.

## Abbreviations

AES: Allelic exchange substrate
GF-AAS: Graphite furnace atomic absorption spectroscopy
OD: Optical density
qRT-PCR: Reverse transcription quantitative PCR
RNS: Reactive nitrogen species
ROS: Reactive oxygen species
TB: Tuberculosis
TM: Transmembrane
WT: Wild type
