# Supplementary figures and images for: CtpB is a plasma membrane copper (I) transporting P-type ATPase of Mycobacterium tuberculosis
Source: Biol Res. 2020 Feb 13;53:6. doi: 10.1186/s40659-020-00274-7 (PMC7017476; doi:10.1186/s40659-020-00274-7)

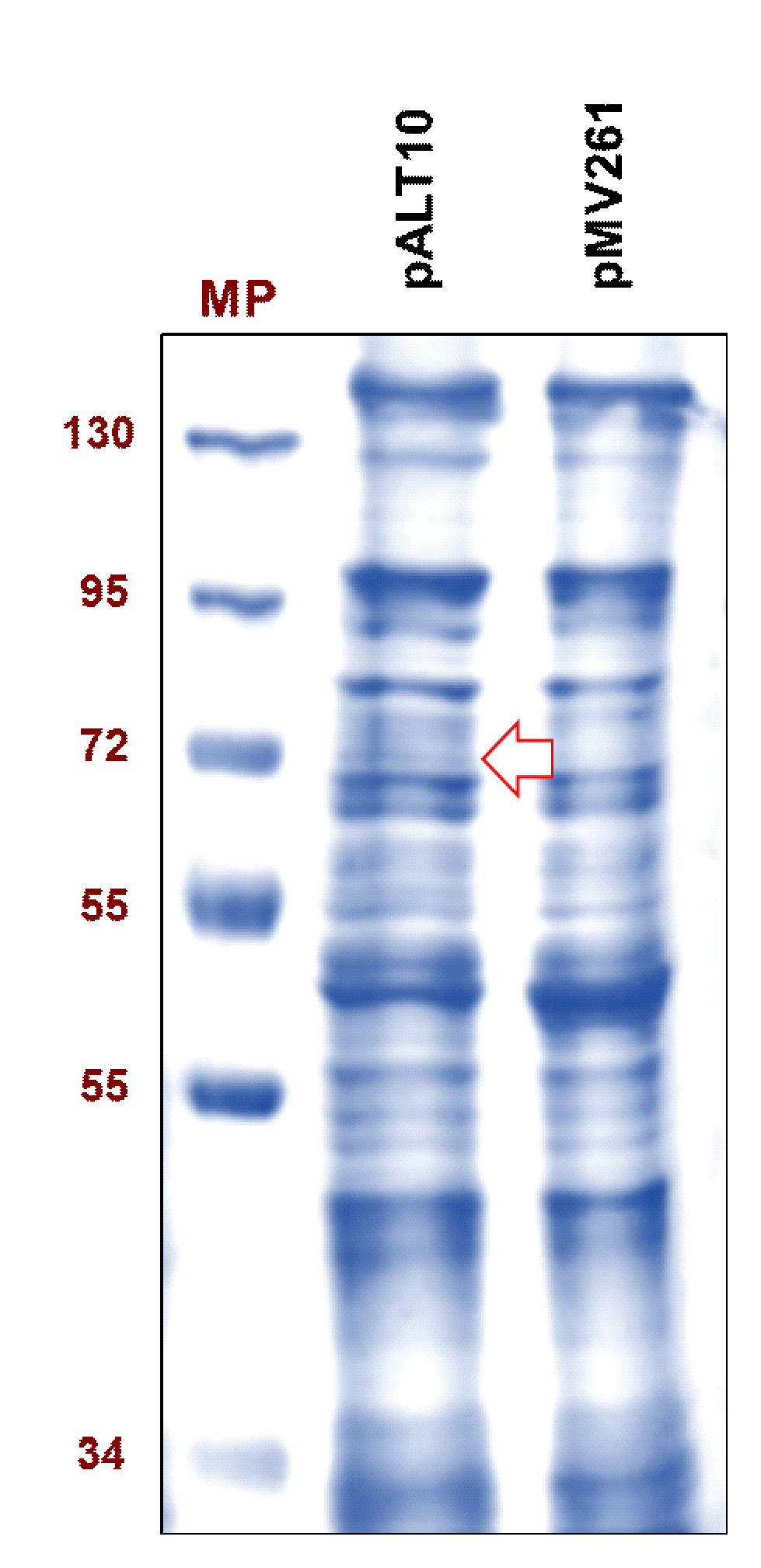

Supplement: Supplementary file 1 — Additional file 1. M. tuberculosis CtpB expression in M. smegmatis plasma membrane. SDS-PAGE analysis of CtpB in the mycobacterial plasma membrane. 12% SDS-PAGE analysis was performed with the membrane fraction of the different cells: pALT10, M. smegmatis expressing CtpB; pMV261, M. smegmatis transformed with the expression vector pMV261 (control without CtpB). The arrow shows a membrane protein of approximately 78.7 kDa corresponding to the molecular weight of the CtpB recombinant protein. [file 40659_2020_274_MOESM1_ESM.jpg]

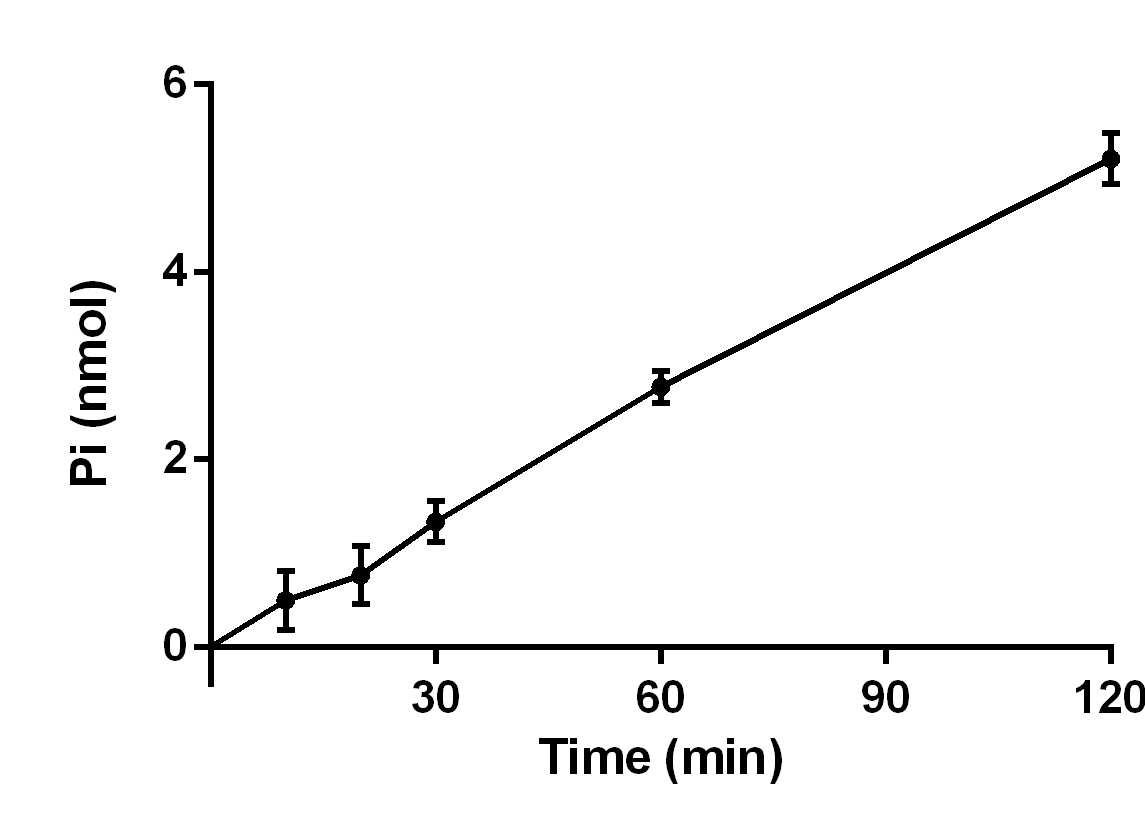

Supplement: Supplementary file 2 — Additional file 2. Enzymatic reaction progress of CtpB. We monitored the Cu+ ATPase reaction progress by measuring the released Pi (nmol) at different enzymatic reaction times. The assay was performed using 10.0 μM Cu+ and enzymatic reactions times between 0 and 60 min. The plotted data correspond to the average released Pi (nmol) ± SEM from three independent experiments. [file 40659_2020_274_MOESM2_ESM.jpg]

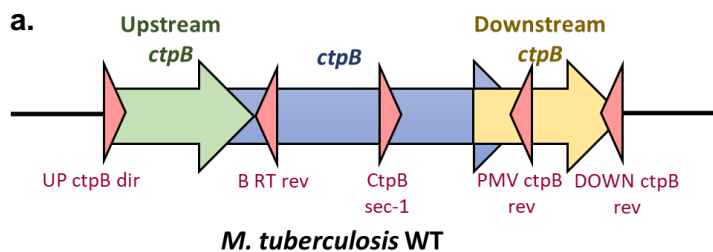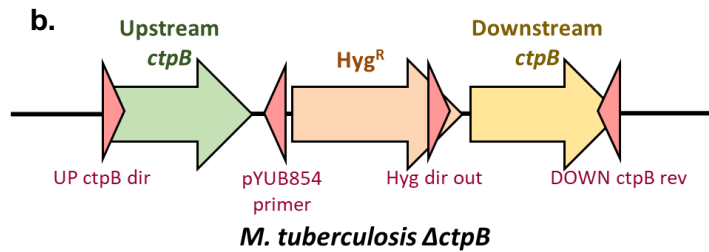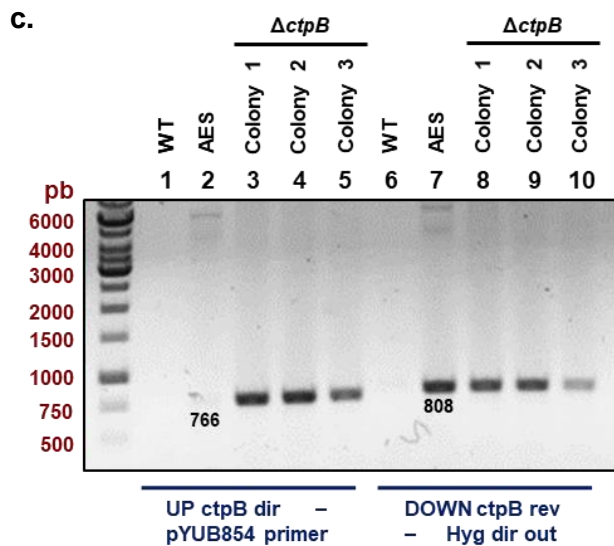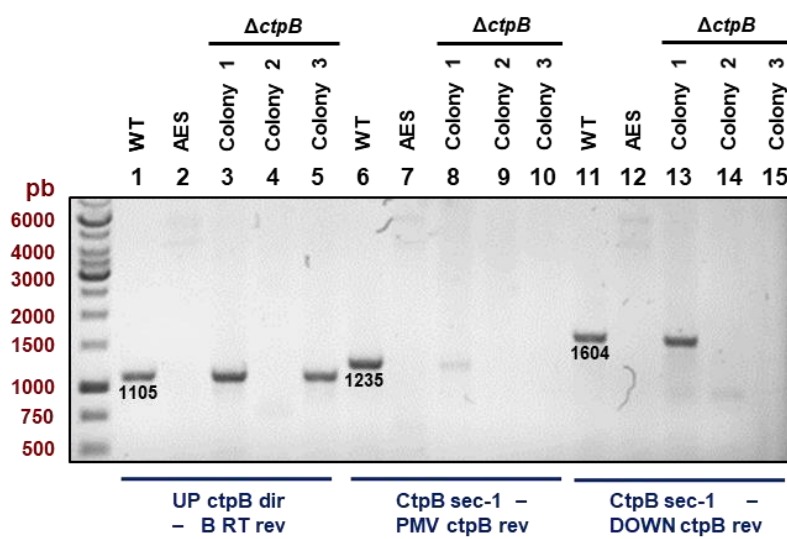

Supplement: Supplementary file 3 — Additional file 3. Disruption of ctpB in M. tuberculosis H37Ra cells. a) Schematic representation of the M. tuberculosis H37Ra ctpB genomic region, indicating the primers used to verify the mutant genotype (negative control). b) Schematic representation of the M. tuberculosis H37Ra genome, where ctpB is interrupted by a Hyg cassette (positive control); the primers used for PCR amplification are shown. c) PCR of the genomic DNA isolated from three possible M. tuberculosis H37Ra ΔctpB colonies using different primer combinations. Genomic DNA from the wild-type strain (WT) and pALT16 plasmid (AES) were used as controls. [file 40659_2020_274_MOESM3_ESM.pdf]
